# Supplementary material for: Towards unified AI-driven fracture mechanics: the extended deep energy method (XDEM)
Source: Nat Commun. 2026 Aug 15;17:8492. doi: 10.1038/s41467-026-76748-1 (PMC13484471; doi:10.1038/s41467-026-76748-1)
Supplement: Supplementary file 3 [file 41467_2026_76748_MOESM3_ESM.zip › pic/Results/Stress_intensity_factor/Cross/comparison_xdem_fem/angle_75/fem_dem_error_uv_3x2_dem_uy.pdf]

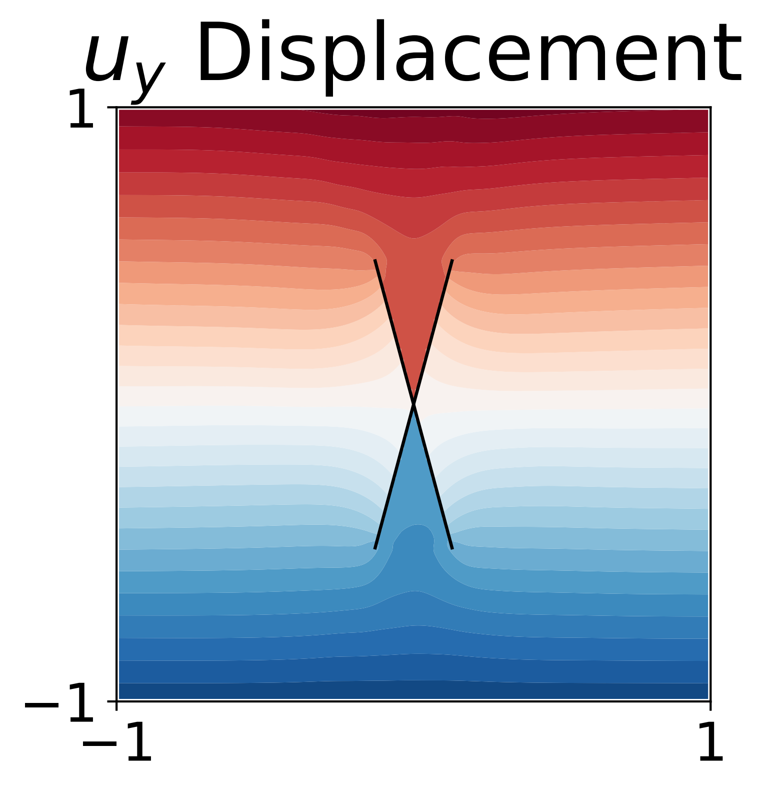

u_y Displacement
1
−1
−1
1
